# Computational simulations of endocrine bone diseases related to pathological glandular PTH secretion using a multi-scale bone cell population model

**DOI:** 10.3389/fbioe.2025.1619276

**Published:** 2025-10-01

**Authors:** Corinna Modiz, Natalia M. Castoldi, Stefan Scheiner, Javier Martínez-Reina, Jose L. Calvo-Gallego, Vittorio Sansalone, Saulo Martelli, Peter Pivonka

**Affiliations:** ^1^ School of Mechanical, Medical and Process Engineering, Queensland University of Technology, Brisbane, QLD, Australia; ^2^ Univ Paris Est Creteil, Univ Gustave Eiffel, CNRS, UMR 8208, MSME, F-94010, Créteil, France; ^3^ Institute for Mechanics of Materials and Structures, TU Wien, Vienna, Austria; ^4^ Departamento de Ingeniería Mecánica y Fabricación, Universidad de Sevilla, Sevilla, Spain

**Keywords:** parathyroid hormone, parathyroid hormone/parathyroid hormone-related protein receptor, bone cell dynamics, disease modeling, pulsatile signal characteristics

## Abstract

**Introduction:**

Bone diseases significantly impact global health by compromising skeletal integrity and quality of life. In disease states linked to parathyroid hormone (PTH) glandular secretion, disrupted PTH patterns typically promote osteoclast proliferation, leading to increased bone resorption.

**Methods:**

While mathematical modeling has proven valuable in analyzing bone remodeling, current bone cell population models oversimplify PTH secretion by assuming constant levels, limiting their ability to represent disorders characterized by variations in PTH pulse characteristics. To address this, we present a novel semi-coupled approach integrating a two-state PTH receptor model with an established bone cell population model. Instead of conventional Hill-type functions, we implement a cellular activity function derived from the receptor model, incorporating pulsatile PTH patterns, cell dynamics, and intracellular communication pathways.

**Results:**

Our numerical simulations demonstrate the model’s capability to reproduce various catabolic bone diseases, providing realistic changes in bone volume fraction over a 1-year period. Notably, while direct implementation of PTH disease progression in the bone cell population model fails to capture diseases only characterized by altered pulse duration and baseline, such as glucocorticoid-induced osteoporosis, our semi-coupled approach successfully models these conditions.

**Discussion:**

This physiologically more realistic approach to endocrine disease modeling offers potential implications for optimizing therapeutic interventions and understanding disease progression mechanisms.

## 1 Introduction

As bone diseases continue to impact global health with serious effects on quality of life, mathematical modeling offers insights into the complex cellular and molecular mechanisms, which control bone remodeling. The objective of the current work is to develop a multi-scale computational model of bone remodeling which can predict bone diseases related to dysfunctional parathyroid gland activity.

The parathyroid gland is responsible for the production of parathyroid hormone (PTH), one of the major hormones in vertebrates besides calcitriol and calcitonin for regulation of calcium homeostasis and bone health (Hernández-Castellano et al., 2020). The pulsatile nature of PTH secretion is a fundamental characteristic shared by many hormones, where pulsatility is believed to modulate target organ responsiveness and shows deviations in disease states as well as circadian and seasonal fluctuations (Chiavistelli et al., 2015). The PTH secretion pattern is characterized by tonic (i.e., constant) and pulsatile components. In healthy subjects, the tonic part of PTH secretion constitutes the majority (70%), whereas approximately 30% is secreted in low-amplitude and high-frequency bursts occurring every 10–20 min, superimposed on the tonic secretion (Chiavistelli et al., 2015). While the exact mechanisms underlying pulsatile hormone secretion are complex and not yet fully understood, the biological importance of PTH pulsatility to bone metabolism is supported by experimental evidence showing that intermittent PTH drug administration produces anabolic effects, whereas continuous administration results in catabolic outcomes (Locklin et al., 2003; Capriani et al., 2012). To understand this complex regulatory mechanism, it is necessary to examine PTH effects at the cellular level.

PTH plays a central role in maintaining calcium homeostasis through a feedback loop: parathyroid cells express calcium-sensing receptors that detect changes in serum calcium levels, with low calcium triggering increased PTH pulse amplitude and frequency, while high calcium results in the opposite effect (Chiavistelli et al., 2015). This creates a regulatory cycle where low serum calcium stimulates PTH secretion, which enhances bone remodeling activity and calcium release, leading to increased serum calcium levels. As calcium levels rise, PTH secretion is reduced and the thyroid releases calcitonin, which lowers the blood calcium level (Sun et al., 2020). Beyond the parathyroid-bone axis, calcium homeostasis involves complex interactions between multiple organ systems, including bone, intra- and extracellular fluid compartments, gut (oral intake), kidney, and the parathyroid gland (Peterson and Riggs, 2010; Sun et al., 2020). Dynamic PTH secretion patterns are fundamental to calcium homeostasis regulation and the pathogenesis of bone diseases.

PTH targets the parathyroid hormone/parathyroid hormone-related protein receptor (PTH/PTHrP type 1 receptor), also commonly known as PTH1R (Cheloha et al., 2015). PTH1R is a G-protein-coupled receptor that regulates skeletal development, bone turnover and mineral homeostasis. PTH1R transduces stimuli from PTH and PTH-related protein (PTHrP) into the interior of target cells (i.e., cells of the osteoblastic lineage) to promote several divergent signaling cascades (Cheloha et al., 2015). Changes in the PTH secretion pattern have been associated with various diseases including primary and secondary osteoporosis, and hyperparathyroidism (Bilezikian et al., 2018; Schaefer, 2000; Harms et al., 1994b; a; Bonadonna et al., 2005), ultimately leading to an imbalanced bone remodeling activity and distorted calcium homeostasis. Simulating bone diseases related to PTH glandular secretion and the corresponding bone cellular activities is the first step in exploring efficient drug treatments.

Osteoporosis (OP) is one of the most frequent musculoskeletal diseases affecting people worldwide (Aibar-Almazán et al., 2022; Salari et al., 2021). OP is characterized by low bone mass and altered bone quality, which ultimately leads to bone fractures. This disease is characterized by imbalanced bone remodeling–the fundamental process that regulates bone homeostasis. In bone remodeling, osteoclastic cells resorb the existing bone matrix, while osteoblastic cells replace the bone matrix by initially forming osteoid, which subsequently gets mineralized. In OP the balance between bone resorption and formation is biased towards resorption with diminished bone formation. Depending on the underlying causes, several types can be distinguished including postmenopausal and senile osteoporosis (Aibar-Almazán et al., 2022). Over the last few decades, a large variety of drugs have been developed, which help combat osteoporosis. The rapid increase in bone biology knowledge has led to the development of mechanobiological pharmacokinetic-pharmacodynamic (PK-PD) models of osteoporosis treatments. As reviewed in Pivonka et al. (2024), these *in silico* models allow predictions beyond bone mineral density (BMD), i.e., bone microdamage and degree of mineralization. Hence, *in silico* trials may serve as complementary tools to experimental studies, potentially contributing to our understanding of drug dosing and combinational treatments, though extensive validation of bone remodeling models across multiple scales remains essential before any clinical application.

Two primary formulations exist for modeling bone cell population dynamics (Cook et al., 2024). The first approach by Komarova et al. (2003) uses power laws where exponent terms represent the accumulated effects of signaling molecules governing both self- and externally-regulated cellular pathways. This results in a relatively small parameter space but creates inherent limitations in interpretability and extensibility. This approach has been further developed for spatio-temporal dynamics (Ryser et al., 2009; 2010). The accumulation of signaling effects makes it difficult to isolate the contributions of individual biomolecular factors on specific cell types. The second approach by Lemaire et al. (2004) employs mass kinetics formulations with explicit Michaelis-Menten and Hill equations for enzyme and ligand binding kinetics. This enables direct identification of how specific signaling factors affect osteoblast and osteoclast concentrations, providing clear biomolecular targets for drug treatments and disease modeling. The explicit modeling of receptor-ligand interactions results in a larger parameter space, but enables studying hormone signaling patterns in healthy and disease states. Thus, we adopt the mass kinetics framework in the present study due to its explicit incorporation of PTH1R receptors expressed on osteoblastic cells and PTH-PTH1R binding kinetics.

Currently no bone disease progression models with links to PTH glandular secretion patterns exist. Endocrine diseases are typically defined by comparing serum levels of endocrine factors with the “normal (or reference) range”. This reference range is used to discern hyper- and hypofunction of respective glands. Dynamic, time-dependent diseases may evolve within the normal range and are characterized by increased or decreased secretory dynamics (Chiavistelli et al., 2015; Schmitt et al., 2005). PTH glandular secretion governs osteoblastic cellular responses in bone and a disturbed function of the parathyroid gland can lead to development of progressive bone diseases. Consequently, the existing knowledge of healthy and pathological PTH glandular secretion patterns need to be incorporated into bone cell population models to accurately describe disease progression.

Schappacher-Tilp et al. (2019) developed a comprehensive mathematical model, which includes the major adaptive mechanisms governing the production, secretion, and degradation of PTH in patients with chronic kidney disease on hemodialysis. This work aimed to investigate the efficacy of parathyroid drugs. The model focused on simulating hemodialysis patients with secondary hyperparathyroidism, but has the potential to be extended and applied to other diseases such as primary hyperparathyroidism or hypo- and hypercalcemia. The ionized calcium concentration–which regulates the parathyroid gland response *via* the calcium sensing receptor–was provided as an input parameter for this model. However, (ionized) calcium concentration depends directly on bone turnover. Consequently, consideration of the bone remodeling process is essential.

Most computational models of bone remodeling are formulated as bone cell population models and include the action of PTH on bone cells in a simplistic manner: constant concentrations of PTH in the central- and/or bone fluid compartment in combination with one-state receptor models (Lemaire et al., 2004; Pivonka et al., 2008; Pivonka and Komarova, 2010; Scheiner et al., 2013; Trichilo et al., 2019). These models simulate catabolic action of PTH on osteoclasts by using a constant PTH concentration as input parameter to mimic both healthy state and particular bone diseases. PTH binds to its receptor PTH1R, expressed on osteoblasts (Datta and Abou-Samra, 2009); the receptor-ligand binding reaction is described by a one-state receptor model. The binding of PTH to its receptor is much faster than a cellular response such as differentiation, proliferation and/or production of ligands of osteoblasts, with binding occurring within minutes (Okazaki et al., 2008) compared to cellular processes that take tens of hours to days (Roberts et al., 1982). Hence, a steady-state assumption is used for solving these binding reactions resulting in a Hill-type equation for the activator/repressor functions, consistent with previous mathematical modeling approaches (Lemaire et al., 2004; Pivonka et al., 2008; Scheiner et al., 2013). The activator/repressor functions are based on receptor occupancy as a function of PTH concentration and total number of receptors expressed on osteoblasts (Datta and Abou-Samra, 2009). In the bone cell population models of Lemaire et al. (2004) and Pivonka et al. (2008), Pivonka and Komarova (2010), the PTH activator and repressor functions influence an intracellular communication pathway, which results in increased osteoclast activity and consequently catabolic bone resorption. While this type of approach is practical and simple to apply for creating catabolic bone remodeling states, it fails to address the link between pathological PTH release patterns and the different observed bone diseases.

Potter et al. (2005) proposed the first computational model to analyze PTH1R kinetics, focusing on the response to constant vs pulsatile dosing patterns of PTH. They introduced a measure of sensitization with values between 1 (highly sensitized) and 0 (desensitized). The study investigated clinically prescribed PTH drug dosing patterns and found a sensitization measure of around 0.9. However, they found a value of 0.89 for healthy glandular PTH secretion patterns. This proximity indicates that the proposed measure of sensitization is not meaningful to use as an activator function for osteoblast response as it is not able to distinguish between anabolic and catabolic actions of PTH.

Recently, Pivonka and co-workers applied the two-state receptor model to PTH1R to analyze the effects of PTH glandular and external dosing patterns on bone cell activity (Martonová et al., 2023). The work focused on clinically observed catabolic bone diseases related to perturbations of PTH glandular secretion. Following the approach proposed by Li and Goldbeter (1989), a cellular osteoblast activity function was developed, which can distinguish various aspects of the stimulation signal including peak dose, time of ligand exposure, and exposure period. Using this formulation, the potential of pharmacological manipulation of the diseased glandular secretion to restore healthy bone, cellular responsiveness *via* clinical approved external PTH injections was investigated. While it was mentioned that the so derived cellular activity function could be linked with a cell population model of bone remodeling, no description on how this could be accomplished was provided.

In this paper we develop a multi-scale bone cell population model based on a two-state receptor model of PTH1R accounting for dynamic PTH secretion patterns. This model is based on our previous work on a (osteoblastic) cellular responsiveness function, which can distinguish different PTH dosing patterns in health and disease (Martonová et al., 2023). We propose a calibration strategy that compares cellular activity values from the two-state receptor model with receptor occupancy values from the one-state model. We solve this problem in a semi-coupled way based on fulfillment of separation of time-scale condition: the two-state receptor model of PTH1R has a characteristic timescale ranging from tens of seconds to tens or hundreds of minutes, while the bone cell population model operates on timescales of hours to tens of days. After model calibration, we validate the model for several glandular disease states and analyze the effect of pulse characteristics on the bone cell response.

## 2 Methods

The bone cell population model (BCPM) describes the temporal behavior of osteoblasts and osteoclasts in various states including their regulation by receptor-ligand interactions. In this section, we present the bone cell population model proposed by Lemaire et al. (2004), and further developed and refined by Pivonka and co-workers (Pivonka et al., 2008; Pivonka and Komarova, 2010; Scheiner et al., 2013; Trichilo et al., 2019). These models provide a robust foundation for our study as they have demonstrated good qualitative agreement with experimental observations from the literature. The Lemaire model successfully reproduces known behaviors of the bone remodeling system, including tight coupling between osteoblasts and osteoclasts, the catabolic effect of continuously elevated PTH and RANKL, the reverse, anabolic effect of increased OPG, and metabolic bone diseases such as estrogen deficiency and glucocorticoid excess. Subsequent developments by Pivonka and co-workers have incorporated bone volume fraction evolution and identified physiologically sensible parameter combinations, while Scheiner’s extension coupled bone cell dynamics with mechanical feedback, reproducing key features of mechanoregulation including postmenopausal osteoporosis progression and mechanical disuse responses. Trichilo et al. further validated the model framework by applying it to ovariectomized rats and comparing bone volume fraction predictions with experimental data. Given our aim to study the effect of the pulsatile glandular secretion pattern of PTH on the bone cell response, here we emphasize the mechanisms through which PTH is integrated and operates in the model by Lemaire et al. A detailed description of the underlying dynamics can be found in the original publication (Lemaire et al., 2004).

The BCPM framework models bone cell populations as averaged concentrations representing the aggregate behavior of bone multicellular units (BMUs) at various stages of the remodeling cycle. While individual BMUs and the respective cells undergo periodic activation, resorption, and formation phases (Kenkre and Bassett, 2018), the model captures the smeared effect of many simultaneously active remodeling sites (Lemaire et al., 2004). In steady state, this approach yields constant average cell concentrations that reflect homeostatic balance rather than the oscillatory dynamics of individual BMUs.

**FIGURE 1:**
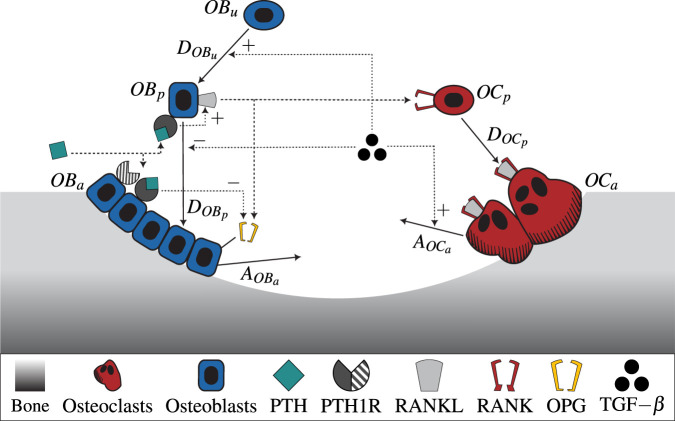
A schematic illustration of the novel bone cell population model including the two-state receptor model for PTH/PTH1R and intracellular communication pathways (TGF- β , RANK-RANKL-OPG). The model includes uncommitted osteoblasts $\left(OB_{u}\right)$ , osteoblast and osteoclast precursors $\left(OB_{p},OC_{p}\right)$ , and active osteoblasts and osteoclasts $\left(OB_{a},OC_{a}\right)$ . The concentrations of $OB_{u}$ and $OC_{p}$ are considered constant and thus included in the respective differentiation rates. The differentiation and apoptosis rate of the corresponding cells are denoted by $D_{OB_{u}},D_{OB_{p}},D_{OC_{p}}$ and $A_{OB_{a}},A_{\mathrm{OCa}}$ . The ligand PTH can bind to its receptor PTH1R in two states: active and inactive, indicated as solid and striped, respectively, in the drawing.

All parameters used in the model including respective description and reference can be found in Supplementary Table S2. Figure 1 shows a schematic illustration of the semi-coupled bone cell population model with the two-state receptor model including intracellular communication pathways and respective parameters.

### 2.1 Mathematical model for bone cell dynamics

The bone cell population model by Lemaire et al. (2004) consists of a system of three ordinary differential equations. It describes the time-dependent behavior of osteoblastic precursor cells $\left(OB_{p}\right)$ , active osteoblasts $\left(OB_{a}\right)$ and active osteoclasts $\left(OC_{a}\right)$ in molar concentrations as

$$\begin{array}{cc} \frac{dOB_{p}}{dt} & =D_{OB_{u}}\cdot \pi_{TGF-\beta }-\frac{D_{OB_{p}}}{\pi_{TGF-\beta }}\cdot OB_{p},\\ \frac{dOB_{a}}{dt} & =\frac{D_{OB_{p}}}{\pi_{TGF-\beta }}\cdot OB_{p}-A_{OB_{a}}\cdot OB_{a},\\ \frac{dOC_{a}}{dt} & =D_{OC_{p}}\cdot \pi_{RANKL}-A_{OC_{a}}\cdot \pi_{TGF-\beta }\cdot OC_{a}. \end{array}$$ (1)

The differentiation rates of uncommitted osteoblasts, osteoblast precursors and osteoclast precursors are denoted by $D_{OB_{u}},D_{OB_{p}}$ and $D_{OC_{p}}$ , respectively; $A_{OB_{a}}$ and $A_{OC_{a}}$ are the apoptosis rates of active osteoblasts and osteoclasts. In line with the source publication (Lemaire et al., 2004), the model assumes that osteoblastic precursor cells $\left(OB_{p}\right)$ do not undergo apoptosis but only differentiate into active osteoblasts, reflecting the biological understanding that once mesenchymal stem cells commit to the osteoblastic lineage, they progress through all differentiation stages (Zhu et al., 2024).

The effects induced by TGF−β are assumed to depend on the concentration of active osteoclasts as

$$\pi_{\mathrm{TGF-\beta}}=\frac{OC_{a}+f_{0}K_{\mathrm{TGF-\beta}}}{OC_{a}+K_{\mathrm{TGF-\beta}}},$$ (2)

with proportionality constant $f_{0}$ and dissociation binding constant $K_{\mathrm{TGF-\beta}}$ for TGF−β and the respective receptor (Lemaire et al, 2004). TGF−β is stored in the bone matrix and released during resorption by active osteoclasts. It promotes $OB_{u}$ -differentiation and $OC_{a}$ -apoptosis, but acts as a repressor on $OB_{p}$ -differentiation. The detailed derivation of $\pi_{\mathrm{TGF-\beta}}$ (Equation 2) can be found in the original publication (Lemaire et al., 2004).

The function $\pi_{\mathrm{RANKL}}$ describes the binding effect of the free ligand RANKL to the corresponding receptor activator nuclear factor κ B (RANK) as

$$\pi_{\mathrm{RANKL}}=\frac{C_{\mathrm{RANK-RANKL}}}{C_{\mathrm{RANK}}},$$ (3)

whereas the molar concentration of RANK-RANKL complexes and RANK are denoted by $C_{\mathrm{RANK-RANKL}}$ and $C_{\mathrm{RANK}}$ , respectively. The receptor-ligand binding triggers osteoclast precursor differentiation. Active osteoblasts secrete osteoprotegerin (OPG), which inhibits this process by binding to RANKL, thereby preventing RANKL-RANK interaction and subsequent osteoclast activation. Further details on $\pi_{\mathrm{RANKL}}$ (Equation 3) are given in the Supplementary Material of this article.

Parathyroid hormone (PTH) influences the RANK-RANKL-OPG pathway catabolically. It binds to its receptor on osteoblasts, increasing RANKL and decreasing OPG concentration. Consequently, more osteoclast precursors are differentiated into active osteoclasts, thus increasing bone resorption. Thus, the ratio of occupied RANK - and consequently $\pi_{\mathrm{RANKL}}$ – depends on the PTH effect, which is quantified by $\pi_{\mathrm{PTH}}$ .

**FIGURE 2:**
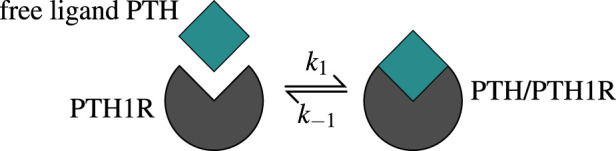
The one-state receptor model describes free ligand PTH binding to its receptor PTH1R on osteoblasts. This binding reaction forms a PTH/PTH1R complex, whereas also the reverse/unbinding reaction is possible. The values of the corresponding kinetic parameters $k_{\pm 1}$ are given in Supplementary Table S2.

The Michaelis-Menten function $\pi_{\mathrm{PTH}}$ describes the fraction of occupied PTH receptors and is derived from a one-state receptor model. The free ligand PTH binds to its receptor PTH1R forming a complex, whereas also the reverse reaction is possible, as shown in Figure 2. The kinetic parameters $k_{1}$ and $k_{-1}$ quantify binding and unbinding tendencies.

The receptor-ligand binding reaches equilibrium long before the bone cells react, which leads to the assumption of a steady state. The function $\pi_{\mathrm{PTH}}$ is defined as

$$\pi_{\mathrm{PTH}}=\frac{C_{\mathrm{PTH}}}{C_{\mathrm{PTH}}+\frac{k_{-1}}{k_{1}}}.$$ (4)

The molar PTH concentration,

$$C_{\mathrm{PTH}}=\frac{C_{\mathrm{PTH}}^{\mathrm{basal}}+C_{\mathrm{PTH}}^{\mathrm{inj}}}{k_{\mathrm{PTH}}},$$ (5)

depends on the basal synthesis rate $\left(C_{\mathrm{PTH}}^{\mathrm{basal}}\right)$ , rate of external PTH injection $\left(C_{\mathrm{PTH}}^{\mathrm{inj}}\right)$ and degradation rate $\left(k_{\mathrm{PTH}}\right)$ . Lemaire et al. (2004) assumed that basal PTH concentration remains constant in both healthy and disease states. When PTH levels are elevated—whether due to disease or injected PTH—this is modeled as a sustained, constant increase from the basal level over a predefined time interval, rather than fluctuating daily. Bone diseases related to the parathyroid gland are characterized by alterations of the pulsatile characteristics, e.g., baseline secretion, pulse height, duration of each pulse and time between successive pulses. This work aims to develop the first bone cell population model that incorporates these characteristics.

### 2.2 Two-state receptor model for pulsatile PTH secretion

PTH1R exhibits multiple conformational states that can be generally classified into active (sensitized) and inactive (desensitized) states, each characterized by distinct signaling responses upon ligand binding (Gardella, 2020; Bisello et al., 2002). The receptor can undergo conformational changes between these states independent of ligand binding, either through covalent modification or simple conformational rearrangement (Wang et al., 2009; Li et al., 2024). The response of osteoblasts to PTH-PTH1R binding depends on the respective conformation state of the receptor, with one state characterized by a shorter response and the other by a prolonged signaling response after receptor-ligand binding.

Martonová et al. (2023) presented a model that accounts for this phenomenon of PTH1R, based on a general formulation of a two-state receptor model by Li and Goldbeter (1989), further detailed in Lauffenburger and Linderman (1993). We describe the essential parts of the model below [a detailed description can be found in Martonová et al. (2023)].

**FIGURE 3:**
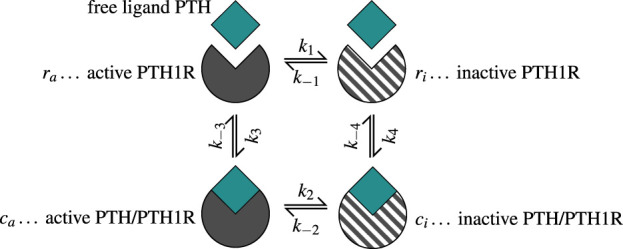
The two-state receptor model describes binding of free ligand PTH to its receptor PTH1R in two states: active/sensitized $\left(r_{a}\right)$ and inactive/desensitized $\left(r_{i}\right)$ . The binding reaction forms complexes in the corresponding states $\left(c_{a},\;c_{i}\right)$ . The concentrations are normalized w.r.t. the total receptor concentration. Binding, unbinding and change of conformation state are governed by the kinetic constants $k_{\pm i}$ with i∈1,2,3,4 given in Supplementary Table S1. Note that the kinetic parameter $k_{\pm 1}$ quantify binding and unbinding tendencies different from those in Figure 2.

Based on the ability of PTH1R to change conformation independent of ligand binding, the model assumes that both receptor conformational states can transform into each other regardless of their binding status (Martonová et al., 2023). The ligand PTH binds to both active (sensitized) and inactive (desensitized) receptors $R_{a},\;R_{i}$ to form complexes in the corresponding states $C_{a},\;C_{i}$ , with reversible unbinding reactions also possible. Both unbound receptors and ligand-receptor complexes can migrate between conformational states, allowing for dynamic transitions between all possible receptor states. All of the described reactions are governed by kinetic constants $k_{\pm j}$ with j∈{1,2,3,4} (Equation 7), as shown in Figure 3.

The receptor and complex concentrations can be summarized in a concentration vector as $C\left(t\right)=\left[R_{a}\left(t\right),C_{a}\left(t\right),C_{i}\left(t\right),R_{i}\left(t\right)\right]$ . The respective time-dependent dynamics are given by a system of differential equations as

$$\frac{dC}{dt}=K\left(L\left(t\right)\right)C{\left(t\right)}^{T}.$$ (6)

The coefficient matrix K describes the kinetics of the receptor-ligand binding depending on the PTH ligand concentration L as

$$K\left(L\right)=\left(\begin{array}{cccc} -k_{1}-k_{3}L & k_{-3} & 0 & k_{-1}\\ k_{3}L & -k_{2}-k_{-3} & k_{-2} & 0\\ 0 & k_{2} & -k_{-2}-k_{-4} & k_{4}L\\ k_{1} & 0 & k_{-4} & -k_{-1}-k_{4}L \end{array}\right).$$ (7)

The kinetic constants are given in Supplementary Table S1.

The ligand concentration *L* (Equation 8) depends on time t , which makes it possible to include the pulsatile PTH pattern. The periodic ligand concentration is approximated as a piece-wise constant function, following the original approach (Martonová et al., 2023) based on the two-state receptor model formulation (Segel et al., 1986; Li and Goldbeter, 1989). This square-wave approximation has been validated against more realistic exponential decay profiles, demonstrating that square-wave stimulation provides a satisfactory approximation to periodic signals with exponential decay (Li and Goldbeter, 1989). This piece-wise constant formulation is commonly employed in bone remodeling models for representing both endogenous hormone pulses (Martonová et al., 2023) and administered drug injection patterns (Trichilo et al., 2019; Lavaill et al., 2020). The periodic ligand concentration is defined as

$$L\left(t\right)=L\left(t+T\right)=\left\{\begin{array}{cc} \gamma_{\mathrm{off}}+\gamma_{on}\; & jT\leq t\leq jT+\tau_{on}\\ \gamma_{\mathrm{off}}\; & jT+\tau_{on}<t<\left(j+1\right)T, \end{array}\right.$$ (8)

for j∈0,1,…,n−1 , where n is the maximum number of periods T during one simulation. The pulse shape is determined by various characteristics: tonic concentration $\gamma_{\mathrm{off}}$ ; pulse height/pulsatile concentration $\gamma_{on}$ ; duration of on-phase $\tau_{on}$ ; and off-phase $\tau_{\mathrm{off}}$ . It holds that $T=\tau_{\mathrm{off}}+\tau_{on}$ .

The dimensionality of system Equation 6 can be reduced to three by normalizing the receptor and complex concentrations relative to total receptor concentration. When expressing the concentrations of active receptors $\left(r_{a}\right)$ , active complexes $\left(c_{a}\right)$ , inactive complexes $\left(c_{i}\right)$ , and inactive receptors $\left(r_{i}\right)$ as normalized values relative to the total receptor concentration, these quantities must satisfy the conservation relation $r_{a}+c_{a}+c_{i}+r_{i}=1$ . This conservation assumption is justified as receptor binding/unbinding dynamics reach steady-state much faster than cellular response timescales (Roberts et al., 1982; Okazaki et al., 2008), and the total receptor number is assumed to be much larger than fluctuations due to receptor production and degradation during the time periods relevant for cellular activity.

The model considers time-dependent ligand concentrations, which we define as pulsatile free PTH based on the glandular secretion pattern. The corresponding cellular activity is given by a linear combination of receptors and complexes in both states as

$$\alpha \left(t\right)=a_{1}r_{a}+a_{2}c_{a}+a_{3}c_{i}+a_{4}r_{i}.$$ (9)

The activity constants $a_{j}$ with j∈{1,2,3,4} determine the weight of the respective concentrations for the cellular activity. The respective values were chosen in line with the original publication Martonová et al. (2023). The activity function (Equation 9) reflects the pulsatile ligand characteristics.

**FIGURE 4:**
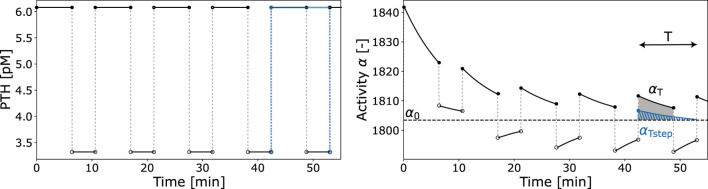
Exemplary PTH pulsatile pattern and resulting activity function α(t) . The integrated activity $\alpha_{T}$ is marked by the shaded area above baseline activity $\alpha_{0}$ (dashed line). The integrated activity $\alpha_{T_{\mathrm{step}}}$ is computed w.r.t. a step increase in PTH (blue). These factors, in conjunction with the period T , constitute the essential parameters required for the calculation of cellular responsiveness $\alpha_{R}$ .

We derive two activity constants based on α(t) : the integrated activity $\alpha_{T}$ and the cellular responsiveness $\alpha_{R}$ . Both capture various characteristics of ligand input and activity output (see Figure 4). The integrated activity $\alpha_{T}$ corresponds to the area under the curve of one activity pulse above baseline $\alpha_{0}$ within one time period T after cellular adaptation. The value of $\alpha_{T}$ is computed by integrating $\alpha \left(t\right)-\alpha_{0}$ over the interval [jT,(j+1)T] for j sufficiently large for the pulses to have reached steady-state. The composite trapezoidal rule is used for numerical integration.

The cellular responsiveness $\alpha_{R}$ depends on $\alpha_{T}$ but incorporates several characteristics of the ongoing stimuli: pulse shape; duration of pulse and off-phase; adaptation of cells to ongoing stimuli. It is defined as

$$\alpha_{R}=\frac{\alpha_{T}}{\alpha_{T_{\mathrm{step}}}}\frac{\alpha_{T}}{T}.$$ (10)

The first factor of Equation 10 relates the integrated activity $\left(\alpha_{T}\right)$ to the integrated activity of a step increase of the same magnitude as the original pulse $\left(\alpha_{T_{\mathrm{step}}}\right)$ . The second factor relates $\alpha_{T}$ to the duration of one pulse and its off-phase (period T ). Figure 4 represents an exemplary pulsatile PTH release pattern, the corresponding activity function α , the derived integrated activity, $\alpha_{T}$ , and remaining determinants of $\alpha_{R}$ such as $\alpha_{T_{\mathrm{step}}}$ and T .

Given that the receptor-ligand binding reaches equilibrium in a very short time (faster than cellular response in the bone cell population model), we propose the use of the activity constant $\alpha_{T}$ and $\alpha_{R}$ to quantify the effect of PTH on osteoblasts. The constants possess the following key features: reflection of the effect of receptor-ligand binding considering two conformation states; consideration of pulsatile, time-dependent behavior of ligand and cellular activity; constant quantification of cellular activity. Thus, we can replace $\pi_{\mathrm{PTH}}$ in the bone cell population model with either $\alpha_{T}$ or $\alpha_{R}$ , to implicitly include pulsatile, time-dependent ligand concentration and the two conformation states of PTH1R.

### 2.3 Bone cell population model and two-state receptor: semi-coupling

We present a semi-coupled approach to integrate the two-state receptor model (Section 2.2) with the bone cell population model (Section 2.1). With this integration, the new bone cell population model includes information incorporated in the cellular activity: characteristics of pulsatile PTH; cellular adaptation to ongoing stimuli; effects of a two-state receptor. The approach involves replacing the Hill-type function $\pi_{\mathrm{PTH}}$ with either the integrated activity $\alpha_{T}$ or the cellular responsiveness $\alpha_{R}$ . Receptor-ligand binding equilibrates considerably faster than bone cell dynamics, which justifies treating Hill-type functions as steady-state constants rather than dynamic functions, as explained in details in the source publication Lemaire et al. (2004). This steady-state assumption was already implicit in the model; therefore, explicitly replacing these functions with constants is both mathematically and conceptually appropriate. Due to differences in magnitude between $\alpha_{T}$ , $\alpha_{R}$ , and $\pi_{\mathrm{PTH}}$ , a scaling approach is necessary. We propose two distinct methods for this scaling.

The first method involves determining scaling parameters ${\tilde{k}}_{R}$ and ${\tilde{k}}_{T}$ based solely on healthy state values,

$${\tilde{k}}_{R}=\frac{\pi_{\mathrm{PTH}}^{\mathrm{healthy}}}{\alpha_{R}^{\mathrm{healthy}}},\;\text{and}\;{\tilde{k}}_{T}=\frac{\pi_{\mathrm{PTH}}^{\mathrm{healthy}}}{\alpha_{T}^{\mathrm{healthy}}},$$ (11)

with

$$\pi_{\mathrm{PTH}}^{\mathrm{healthy}}=\frac{C_{\mathrm{PTH}}^{\mathrm{basal}}}{C_{\mathrm{PTH}}^{\mathrm{basal}}+\frac{k_{-1}}{k_{1}}},$$ (12)

whereas Equation 12 refers to the original Hill-type function for healthy state. The corresponding parameter values are given in Supplementary Table S2. The constants $\alpha_{T}^{\mathrm{healthy}}$ and $\alpha_{R}^{\mathrm{healthy}}$ are computed from Equation 10 and preceding formulation based on the healthy pulse characteristics (Table 1). These parameters are subsequently applied across all disease states. The kinetic parameter $k_{\pm 1}$ quantify binding and unbinding tendencies of the one-state receptor model (Figure 2).

**TABLE 1:** Parameters for healthy and disease states for PTH concentration.

State	$\gamma_{\text{off}}$ [nM]	$\gamma_{\text{on}}$ [nM]	$\tau_{\text{on}}$ [min]	$\tau_{\text{off}}$ [min]	T [min]
Healthy	$3.32\cdot 10^{-3}$	$2.76\cdot 10^{-3}$	6.4	4.2	10.6
HPT	$1.38\cdot 10^{-2}$	$9.77\cdot 10^{-3}$	7.6	3.5	11.1
OP	$3.32\cdot 10^{-3}$	$1.70\cdot 10^{-3}$	5.2	24.6	29.8
PMO	$2.99\cdot 10^{-3}$	$2.48\cdot 10^{-3}$	6.4	4.2	10.6
HyperC	$8.30\cdot 10^{-4}$	$3.31\cdot 10^{-4}$	9.41	6.18	15.59
HypoC	$8.53\cdot 10^{-3}$	$3.62\cdot 10^{-2}$	3.27	2.14	5.41
GIO	$1.59\cdot 10^{-3}$	$4.83\cdot 10^{-3}$	6.08	3.99	10.07

The pulse shape is determined by the off-phase, $\gamma_{\text{off}}$ , and the height of the pulse, $\gamma_{\text{on}}$ . The sum of the duration of off-phase, $\tau_{\text{off}}$ , and on-phase, $\tau_{\text{on}}$ , is the period T . The values are taken from Martonová et al. (2023), where disease states were computed using relative changes from healthy people based on experimental data. The considered disease states are Hyperparathyroidism (HPT), Osteoporosis (OP), Postmenopausal Osteoporosis (PMO), Hypercalcemia (HyperC), Hypocalcemia (HypoC), Glucocorticoid-induced Osteoporosis (GIO).

The second method is based on a comprehensive optimization approach across all states, determining parameters $k_{R}$ and $k_{T}$ through minimization,

$$\underset{k_{R}}{\min}\overset{7}{\underset{i=1}{\sum }}{\left(k_{R}\cdot \alpha_{R}^{i}-\pi_{\mathrm{PTH}}^{i}\right)}^{2}\;\text{and}\;\underset{k_{T}}{\min}\overset{7}{\underset{i=1}{\sum }}{\left(k_{T}\cdot \alpha_{T}^{i}-\pi_{\mathrm{PTH}}^{i}\right)}^{2}.$$ (13)

Here, index i represents all seven physiological states detailed in Table 1. This optimization was implemented using Python’s SciPy library, using the default Broyden-Fletcher-Goldfarb-Shanno (BFGS) quasi-Newton method.

To establish comparable disease states between the pulsatile two-state receptor model (Section 2.2) and the constant-secretion reference model (Section 2.1), we introduce a scaling parameter for each disease given in Table 1,

$$a_{\mathrm{PTH}}^{\mathrm{disease}}=\frac{\max\left(C_{\mathrm{PTH,disease}}^{\mathrm{basal}}\right)}{\max\left(C_{\mathrm{PTH,healthy}}^{\mathrm{basal}}\right)}=\frac{\gamma_{\mathrm{off}}^{\mathrm{Disease}}+\gamma_{on}^{\mathrm{Disease}}}{\gamma_{\mathrm{off}}^{\mathrm{Healthy}}+\gamma_{on}^{\mathrm{Healthy}}}.$$ (14)

**FIGURE 5:**
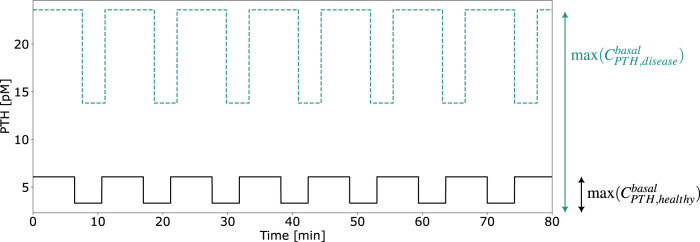
Exemplary computation of the calibration parameter $a_{\mathrm{PTH}}^{\mathrm{disease}}$ for healthy and disease states defined by Equation (14).

This parameter, illustrated in Figure 5, modifies the PTH concentration equation (Equation 5) for disease modeling in the reference model,

$$C_{\mathrm{PTH}}=\frac{a_{\mathrm{PTH}}^{\mathrm{disease}}C_{\mathrm{PTH}}^{\mathrm{basal}}}{k_{\mathrm{PTH}}},$$ (15)

whereas $C_{\mathrm{PTH}}^{\mathrm{basal}}$ refers to the basal concentration of the original model formulation. Following these scaling approaches, $\pi_{\mathrm{PTH}}$ (Equation 4) is replaced with either ${\tilde{k}}_{R}\alpha_{R}$ , $k_{R}\alpha_{R}$ , ${\tilde{k}}_{T}\alpha_{T}$ or $k_{T}\alpha_{T}$ to analyze the effect of the different constants and scaling approaches. The activity constants are computed independently (Section 2.2), reflecting the faster equilibration of PTH-PTH1R binding compared to bone cell response dynamics. Consequently, the RANK occupancy ratio in the bone cell population model becomes dependent on these scaled activities for both healthy and disease states.

To further validate and compare the model approaches, we analyze the temporal evolution of bone volume fraction, $f_{bm}$ , describing the bone volume per total volume. Following Scheiner et al. (2013), we define the change in bone volume fraction with time as

$$\frac{df_{bm}}{dt}=k_{\mathrm{form}}\cdot OB_{a}\left(t\right)-k_{\mathrm{res}}\cdot OC_{a}\left(t\right)$$ (16)

where $k_{\mathrm{form}}$ and $k_{\mathrm{res}}$ represent the formation and resorption rates (bone volume formed and resorbed per unit cell concentration per unit time), respectively. This equation captures how bone volume fraction changes over time as a result of the competing processes of bone formation by active osteoblasts $OB_{a}$ and bone resorption by active osteoclasts $OC_{a}$ . Integration of this equation yields the temporal bone volume fraction profiles. In homeostasis, an equal fraction of bone volume is formed and resorbed. Thus, knowing the bone formation rate at a particular bone site (femoral neck, lumbar vertebra, radius) in homeostasis, the resorption rate can be computed as

$$k_{\mathrm{res}}=\frac{k_{\mathrm{form}}OB_{a}^{ss}}{OC_{a}^{ss}},$$ (17)

based on the steady-state concentration of osteoblasts $OB_{a}^{ss}$ and osteoclasts $OC_{a}^{ss}$ .

### 2.4 Analysis of pulse characteristics

With the new model formulation, we can analyze the effect of different pulse characteristics on bone cell dynamics. The objective is to compare the magnitude of the activity constants of disease states to the healthy state with varying duration of pulse on- and off-phase, $\tau_{on}$ and $\tau_{\mathrm{off}}$ , respectively. The period T remains fixed to the physiological value according to Table 1 and it must hold that $T=\tau_{on}+\tau_{\mathrm{off}}$ . To maintain physiological relevance, $\tau_{\mathrm{off}}\geq 0$ and $\tau_{on}\geq 0$ must be fulfilled. For this analysis, the phases $\tau_{on}$ and $\tau_{\mathrm{off}}$ may take every value possible that fulfills the above requirements. Thus, the on- and off-phases are determined according to

$$\tau_{on}\in \left[0,T\right],\;\tau_{\mathrm{off}}=T-\tau_{on},$$ (18)

whereas the same results can be obtained if $\tau_{on}$ and $\tau_{\mathrm{off}}$ are switched in the above formulation as both cover the entire range of 0 to T .

## 3 Results

In this section, we describe the results of the calibration strategy given in Section 2.3, followed by the cellular activity and bone cell dynamics of the final, semi-coupled model. Finally, we demonstrate the effect of selected pulse characteristics on bone cell dynamics.

### 3.1 Calibration of Hill-type function and activity constants

We follow the approach given in Section 2.3 to include pulsatile PTH and PTH1R in two conformation states in the bone cell population model. We solve both Equation 11 and the minimization problem in Equation 13 to consider the different orders of magnitude of the Hill-type function $\pi_{\mathrm{PTH}}$ , integrated activity $\alpha_{T}$ and cellular responsiveness $\alpha_{R}$ .

**FIGURE 6:**
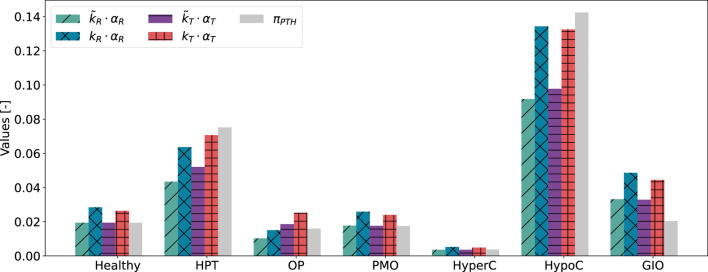
Results of scaling approaches for cellular responsiveness, $\alpha_{R}$ , integrated activity, $\alpha_{T}$ , and formerly used Hill-type function, $\pi_{\mathrm{PTH}}$ , for healthy and disease states: hyperparathyroidism (HPT), osteoporosis (OP), postmenopausal osteoporosis (PMO), hypercalcemia (HyperC), hypocalcemia (HypoC), glucocorticoid-induced osteoporosis (GIO). The calibration constants $k_{R},k_{T}$ are identified considering all disease states (Equation 13), the scaling constants ${\tilde{k}}_{R},{\tilde{k}}_{T}$ only consider the healthy state (Equation 11).

Both approaches to achieve comparable order of magnitude between $\alpha_{T}$ , $\alpha_{R}$ and $\pi_{\mathrm{PTH}}$ result in similar values for the scaling parameters (Table 2). The aligned activity constants ${\tilde{k}}_{T}\alpha_{T},{\tilde{k}}_{R}\alpha_{R}$ equal the formerly used function $\pi_{\mathrm{PTH}}$ for the healthy state and show only a small deviation for postmenopausal osteoporosis and hypercalcemia (Figure 6). Hyperparathyroidism, hypocalcemia and glucocorticoid-induced osteoporosis show larger deviations.

**TABLE 2:** Results of scaling of $\alpha_{R}$ and $\alpha_{T}$ to achieve same order of magnitude as formerly used $\pi_{\mathrm{PTH}}$ .

Scaling parameter	Parameter value [-]	ASE [-]	RMSE [-]	Relative RMSE [-]
${\tilde{k}}_{R}$	2.07·10^−2^	—	—	—
${\tilde{k}}_{T}$	5.29·10^−4^	—	—	—
$k_{R}$	3.03·10^−2^	1.15·10^−3^	1.28·10^−2^	9.22·10^−2^
$k_{T}$	7.17·10^−4^	8.72·10^−4^	1.12·10^−2^	8.04·10^−2^

We identified the parameters ${\tilde{k}}_{R},{\tilde{k}}_{T}$ for the healthy state and $k_{R},k_{T}$ for all seven states (healthy and six disease states) using a minimization approach. The absolute squared error (ASE) refers to the result of the minimization problem (Equation 13). After identification of the scaling parameters using minimization, we calculate root mean square error (RMSE) and RMSE relative to the range of $\pi_{\mathrm{PTH}}$ .

Despite relying on a single identified parameter across all states, both activity constants $\alpha_{T}$ and $\alpha_{R}$ show consistent trends with the Hill-type function $\pi_{\mathrm{PTH}}$ (Figure 6). This consistency is also reflected in the small absolute error (ASE) for both minimization problems, which can be found in a range of $10^{-3}$ to $10^{-4}$ for both $\alpha_{T}$ and $\alpha_{R}$ (Table 2). The root mean square error (RMSE) relative to the range of Hill-type functions for the given states shows that the typical error is about 9% of the full span of $\pi_{\mathrm{PTH}}$ for cellular responsiveness and 8% for integrated activity. The closest match is found for hypercalcemia, whereas $\alpha_{T}$ and $\alpha_{R}$ show the largest deviation from $\pi_{\mathrm{PTH}}$ for glucocorticoid-induced osteoporosis.

### 3.2 Cellular activity and bone cell dynamics

**FIGURE 7:**
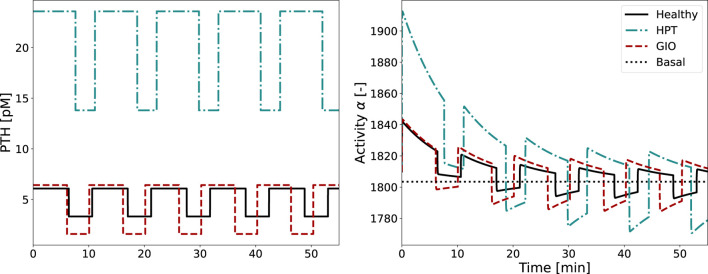
Glandular PTH secretion pattern and corresponding activity function α(t) for healthy state (solid line), hyperparathyroidism (HPT, dash-dotted line) and glucocorticoid-induced osteoporosis (GIO, dashed line). The basal activity is included as dotted line.

The cellular activity function α(t) is computed for a PTH pattern in healthy and disease state according to Table 1. Figure 7 shows that for hyperparathyroidism (HPT), the tonic secretion increases more than double the maximum of healthy PTH concentration. Not only does HPT change the amount of PTH secretion, but also the duration of PTH secretion. Compared with a healthy state, the characteristic pulse lasts longer and the off-phase is shorter, ultimately resulting in a longer period T (on- and off-phase). In glucocorticoid-induced osteoporosis (GIO), where PTH shows a lower basal secretion, its pulses reach almost the same maximum concentration as in healthy controls. This creates a larger relative amplitude of the pulses in GIO, despite similar absolute peak values.

The activity function α(t) reflects every change of the pulsatile PTH pattern. As shown in Figure 7, the cellular activity of HPT also has a longer period T and on-phase $\tau_{on}$ of the pulse compared to the healthy state. The pulse height is elevated, whereas the activity pulse below as well as above baseline activity $\alpha_{0}$ is longer than the healthy activity. For GIO, the activity pulse is longer compared to the healthy pattern, resulting in a higher fraction above baseline activity.

In both healthy and diseased conditions, cellular adaptation to the stimulus occurs during the first two periods, with all subsequent pulses looking identical. As the integrated activity $\alpha_{T}$ and cellular responsiveness $\alpha_{R}$ are computed based on the activity function α(t) , they also reflect any alterations of pulse characteristics. For example, both $\alpha_{T}$ and $\alpha_{R}$ increased noticeably for HPT, reflecting both prolonged period and activity pulse (see Table 3). One pulse remains longer above baseline and has a higher maximum value compared to the healthy state, leading to an increase in $\alpha_{T}$ and $\alpha_{R}$ . For GIO, both $\alpha_{T}$ and $\alpha_{R}$ are almost doubled, reflecting the higher maximum activity.

**TABLE 3:** Values of integrated activity, $\alpha_{T}$ , and cellular responsiveness, $\alpha_{R}$ , for healthy state and all included disease states: hyperparathyroidism (HPT), osteoporosis (OP), postmenopausal osteoporosis (PMO), hypercalcemia (HyperC), hypocalcemia (HypoC), glucocorticoid-induced osteoporosis (GIO).

Activity constant	Healthy	HPT	OP	PMO	HyperC	HypoC	GIO
$\alpha_{T}$	36.6479	98.3844	35.1993	33.2851	6.7742	184.7505	62.0019
$\alpha_{R}$	0.9376	2.1014	0.4973	0.8550	0.1741	4.4375	1.6052

**FIGURE 8:**
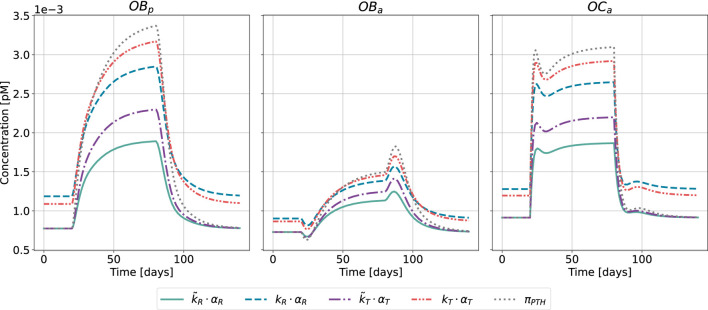
Bone cell dynamics resulting from the novel approach of the bone cell population model by semi-coupling with a two-state receptor model. The concentrations of osteoblast precursors, $OB_{p}$ , active osteoblasts, $OB_{a}$ , and active osteoclasts, $OC_{a}$ , are shown for healthy state and hyperparathyroidism (HPT). The instantaneous disease state is defined for a chosen time interval, t∈[20,80] . Each cell concentration is plotted for different approaches: scaling parameter identified only for healthy state $\left({\tilde{k}}_{R}\cdot \alpha_{R},{\tilde{k}}_{T}\cdot \alpha_{T}\right)$ ; scaling parameter identified for all states $\left(k_{R}\cdot \alpha_{R},k_{T}\cdot \alpha_{T}\right)$ ; reference model $\left(\pi_{\mathrm{PTH}}\right)$ .

**FIGURE 9:**
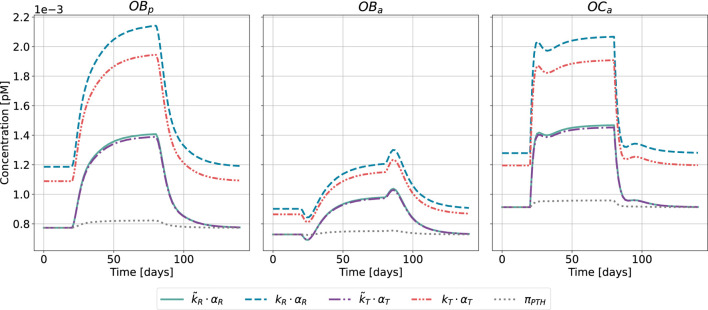
Bone cell dynamics resulting from the novel approach of the bone cell population model by semi-coupling with a two-state receptor model. The concentrations of osteoblast precursors, $OB_{p}$ , active osteoblasts, $OB_{a}$ , and active osteoclasts, $OC_{a}$ , are shown for healthy state and glucocorticoid-induced osteoporosis (GIO). The instantaneous disease state is defined for a chosen time interval, t∈[20,80] . Each cell concentration is plotted for different approaches: scaling parameter identified only for healthy state $\left({\tilde{k}}_{R}\cdot \alpha_{R},{\tilde{k}}_{T}\cdot \alpha_{T}\right)$ ; scaling parameter identified for all states $\left(k_{R}\cdot \alpha_{R},k_{T}\cdot \alpha_{T}\right)$ ; reference model $\left(\pi_{\mathrm{PTH}}\right)$ . The reference model - without taking pulsating information into account - hardly reflects the effect of GIO.

After scaling to achieve same order of magnitude, $\pi_{\mathrm{PTH}}$ can be replaced with either $k_{T}\cdot \alpha_{T}$ , ${\tilde{k}}_{T}\cdot \alpha_{T}$ , $k_{R}\cdot \alpha_{R}$ or ${\tilde{k}}_{R}\cdot \alpha_{R}$ for healthy and disease state. The healthy state corresponds to a steady state of the model described by Equation 1. While transition from a healthy state to a pathological state in terms of PTH glandular secretion pattern might take some time (i.e., months to years), in numerical simulations the switch from healthy to disease is set instantaneously at a defined time point, as shown in Figure 8 for the case of HPT and in Figure 9 for GIO.

The results of the semi-coupled bone cell population model for HPT as disease state are shown in Figure 8. The cellular concentrations for $OB_{p}$ , $OB_{a}$ and $OC_{a}$ have consistent dynamics across both the cellular responsiveness ( ${\tilde{k}}_{R}\cdot \alpha_{R}$ , $k_{R}\cdot \alpha_{R}$ ) and integrated activity $\left({\tilde{k}}_{T}\cdot \alpha_{T},k_{T}\cdot \alpha_{T}\right)$ compared to the previously used Hill-type function $\left(\pi_{\mathrm{PTH}}\right)$ . All five approaches demonstrate similar characteristic patterns, the main difference is the magnitude of the cellular concentrations in steady-state and disease case for calibration considering all states $\left(k_{R},k_{T}\right)$ . The steady-state is equivalent for ${\tilde{k}}_{R}\cdot \alpha_{R},{\tilde{k}}_{T}\cdot \alpha_{T}$ and $\pi_{\mathrm{PTH}}$ in line with the identification of the scaling parameters based on only the healthy state.

Regarding the relative distance of $OB_{p}$ to $OB_{a}$ and $OC_{a}$ , respectively, the concentration of $OB_{p}$ is closer to $OB_{a}$ during the steady-state (before and after disease state) using the original model formulation compared to the second calibration approach $\left(k_{R},k_{T}\right)$ . The $OB_{p}$ curve starts and levels off closer to $OC_{a}$ concentration when using the activity constants. The homeostasis values of all three cell types are different for the model using either $\pi_{\mathrm{PTH}}$ , $k_{T}\alpha_{T}$ or $k_{R}\alpha_{R}$ to quantify the PTH effect. After the immediate switch to a disease state, the increase in concentration varies across all cell types, models and scaling approaches. The original formulation shows the highest peak of all cell concentrations, whereas the cellular responsiveness, ${\tilde{k}}_{R}\alpha_{R}$ , results in the lowest increase.

The results of the semi-coupled bone cell population model for GIO as disease state are shown in Figure 9.

The bone cell concentrations show different dynamics for the original model formulation and the novel semi-coupled approach. The original model formulation results in cell concentrations in a range of 1⋅10^−4^ and reduced dynamics. The onset of the disease state is barely visible in the time interval between 20 and 80 days. In contrast to the formerly used $\pi_{\mathrm{PTH}}$ , the change from homeostasis to GIO is clearly reproduced in the novel approach. The maximum concentration of $OB_{p}$ and $OC_{a}$ is found close to 2⋅10^−3^ when using either cellular responsiveness $k_{R}\cdot \alpha_{R}$ or integrated activity $k_{T}\cdot \alpha_{T}$ . The second scaling approach $\left({\tilde{k}}_{R}\cdot \alpha_{R},{\tilde{k}}_{T}\cdot \alpha_{T}\right)$ results in lower cell concentrations after onset of the disease state compared to the alternative approach.

**FIGURE 10:**
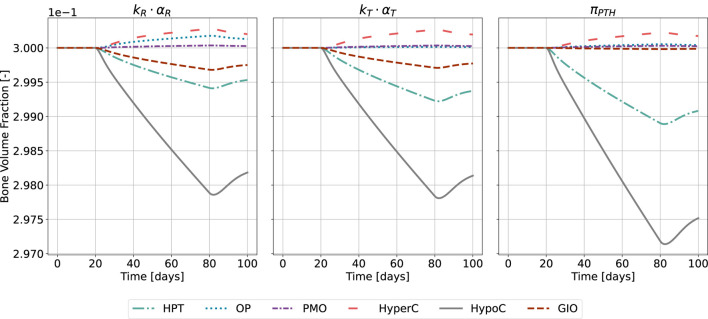
Bone volume fraction resulting from the novel approach of the bone cell population model by semi-coupling with a two-state receptor model. The scaling parameter is identified for all states $\left(k_{R}\cdot \alpha_{R},k_{T}\cdot \alpha_{T}\right)$ and compared to the reference model $\left(\pi_{\mathrm{PTH}}\right)$ . The instantaneous disease state is defined for a chosen time interval, t∈[20,80] . The bone volume fraction evolution is shown for hyperparathyroidism (HPT), osteoporosis (OP), postmenopausal osteoporosis (PMO), hypercalcemia (HyperC), hypocalcemia (HypoC), glucocorticoid-induced osteoporosis (GIO). The initial trabecular bone volume fraction is chosen as 0.3.

This is reflected in the change of bone volume fraction during disease state (Figure 10). The initial bone volume fraction is chosen as 0.3 for trabecular bone (Ding et al., 1999); the temporal evolution is described by Equations 16, 17. For the sake of conciseness and due to the comparable outcomes of both approaches, we present only the results of the calibration method accounting for all disease states and the formerly used $\pi_{\mathrm{PTH}}$ as reference. For GIO, the novel model approach using both cellular responsiveness $\alpha_{R}$ and integrated activity $\alpha_{T}$ results in a loss of bone volume between 0.6% and 0.7% after 60 days of disease simulation. The loss of bone volume in the original model formulation is not significant for this disease state - in line with the reduced bone cell response (Figure 9). The highest catabolic effect during simulation of GIO is observed for $k_{R}\cdot \alpha_{R}$ .

The catabolic effect of hyperparathyroidism (HPT) and hypocalcemia (HypoC) are reflected in all model approaches and highest for the original formulation (Figure 10). Osteoporosis (OP), hypercalcemia (HyperC) and postmenopausal osteoporosis (PMO) show minor positive or no deviations from homeostasis across all approaches.

### 3.3 Pulse characteristics and homeostasis

Following the methodology presented in Section 2.4, we vary on- and off-phase of the pulse whereas all other characteristics remain fixed to physiologically healthy values. On- and off-phase are restricted to sum to the period T (Equation 18).

**FIGURE 11:**
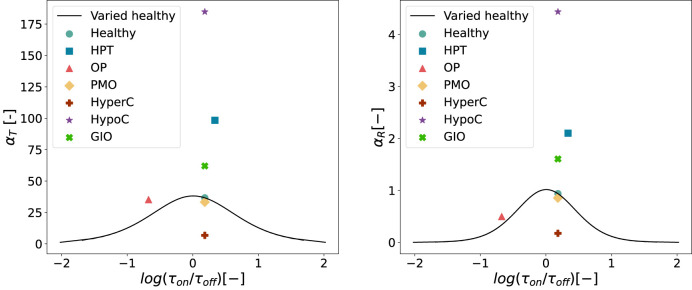
Values of $\alpha_{T}$ and $\alpha_{R}$ for the healthy state depending on/off ratio. Physiological healthy and disease states (hyperparathyroidism (HPT), osteoporosis (OP), postmenopausal osteoporosis (PMO), hypercalcemia (HyperC), hypocalcemia (HypoC), glucocorticoid-induced osteoporosis (GIO)) are also included in the plot for comparison. Left: On/off-ratio variations with *α*_*T*_. Right: On/off-ratio variations with *α*_*R*_.

Figure 11 shows the resulting values of integrated activity $\alpha_{T}$ and cellular responsiveness $\alpha_{R}$ w.r.t. the ratio of on- and off-phase. The curve is symmetrical around the point where $\tau_{on}$ equals $\tau_{\mathrm{off}}$ , represented by the logarithmic ratio $\log\left(\frac{\tau_{on}}{\tau_{\mathrm{off}}}\right)=0$ . The maximum of both $\alpha_{T}$ and $\alpha_{R}$ at this point corresponds to the highest catabolic response with healthy tonic and pulsatile PTH. Both activity constants are close to zero when $\tau_{on}$ is much larger than $\tau_{\mathrm{off}}$ or the other way around.

For both $\alpha_{T}$ and $\alpha_{R}$ , the healthy value (cyan circle) appears near the maximum of both curves, occurring at a slightly positive $\log\left(\tau_{on}/\tau_{\mathrm{off}}\right)$ ratio (see Figure 11). Hypocalcemia (purple star) exhibits the highest response in both metrics, which stand distinctly above other pathological conditions. Most disease states cluster around similar $\tau_{on}/\tau_{\mathrm{off}}$ ratios, indicating a common temporal pattern in PTH and receptor dynamics across different pathological conditions. However, osteoporosis (orange triangle) presents a notable exception to this trend. Not only does it occur at a different $\tau_{on}/\tau_{\mathrm{off}}$ ratio compared to other conditions, but it also shows the largest difference when comparing $\alpha_{R}$ and $\alpha_{T}$ responses.

**FIGURE 12:**
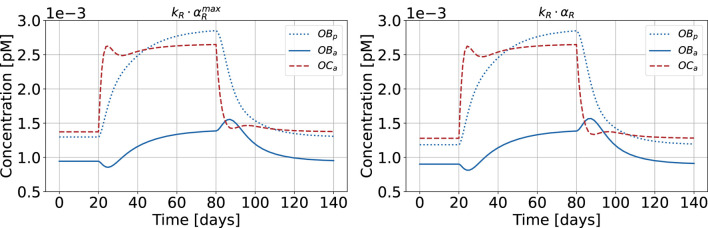
Bone cell population model with maximum cellular responsiveness $\alpha_{R}$ for healthy state and HPT as disease state (left). The results using non-optimized cellular responsiveness $\alpha_{R}$ for healthy state are shown as comparison (right). The concentrations of active osteoblasts, $OB_{a}$ , and osteoblast precursors, $OB_{p}$ , are shown.

Figure 12 shows the results of the bone cell population model for healthy state and HPT when replacing $\pi_{\mathrm{PTH}}$ not only with the calibrated cellular responsiveness, but with the maximum cellular responsiveness $\alpha_{R}^{\max}$ resulting from the pulse characteristics $\tau_{on}^{\max}$ and $\tau_{\mathrm{off}}^{\max}$ for healthy state (see Figure 11). This results in elevated baseline concentrations across all cell populations ( $OB_{p}$ , $OB_{a}$ , and $OC_{a}$ ), while showing a proportionally lower catabolic jump in hyperparathyroidism. We selected the cellular responsiveness $\alpha_{R}$ instead of $\alpha_{T}$ because its response curve is steeper around the maximum. This provides better discrimination of the effect of different pulse characteristics, as evidenced by the distinct positioning of, for example, osteoporosis (see Figure 11). The temporal dynamics remain consistent between both configurations.

## 4 Discussion

This study presents a novel approach to simulating bone disease progression related to the human parathyroid gland. We analyzed alterations of parathyroid hormone release patterns within the framework of bone cell population models (BCPMs) – a central step toward more physiologically realistic modeling of disease progression and accurate pharmacokinetics-pharmacodynamic (PK-PD) models (Pivonka et al., 2024). While traditional BCPMs treat hormone concentrations as constants, we demonstrated that dynamic hormonal patterns contain valuable information about disease states that is lost when complex temporal dynamics are simplified to averaged reference constants. Indeed, it is not clear how changes in dynamic hormonal release patterns could be implemented in the mechanistic BCPM framework. To address this question, we proposed a semi-coupled approach of a two-state PTH-PTH1R receptor model with the original bone cell population model of Lemaire et al. (2004) incorporating the bone volume fraction balance equation (Pivonka et al., 2008). Despite increased complexity, the cell concentration dynamics remain qualitatively similar to the original model formulation, demonstrating the robustness across different model variants (Figures 8, 9). Extension to more complex BCPMs (Pivonka et al., 2008; Pivonka and Komarova, 2010; Scheiner et al., 2013; Trichilo et al., 2019) is straightforward.

The model successfully reproduces key bone cell responses including increased osteoclast activity during disease states and time-delayed osteoblast response to catabolic osteoclast action. We showed that catabolic responses, traditionally obtained by increasing the PTH activator function $\left(\pi_{\mathrm{PTH}}\right)$ , can now be achieved by replacing $\pi_{\mathrm{PTH}}$ with the calibrated cellular responsiveness $\alpha_{R}$ or integrated activity $\alpha_{T}$ from the two-state PTH-PTH1R model. While the latter quantities were previously given theoretical bone-related interpretations in the two-state receptor model (Martonová et al., 2023), our study represents their first implementation within an actual bone cell population modeling framework.

Our analysis of single pulse characteristics (Section 2.4) shows that maximum cell response occurs at equal duration of on- and off-phase. This demonstrates the inherent trade-offs between pulse height and width: very long PTH pulses result in extended but low activity due to receptor desensitization, while short pulses produce intense but brief responses. The highest catabolic response–quantified by maximal integrated activity $\alpha_{T}$ and cellular responsiveness $\alpha_{R}$ – occurs when on- and off-phases are of equal duration, balancing pulse height and width while allowing for receptor resensitization between pulses.

We compared our novel approaches with the original BCPM formulation to evaluate the importance of including pulsatile PTH dynamics. We implemented disease states in the original model using a “scaling parameter” that reflects how diseases would be modeled as constant PTH elevations rather than capturing the full pulsatile patterns. The parameter is the ratio of maximum PTH concentration in disease and healthy state (Equation 14), and we multiplied it with the baseline PTH concentration of the BCPM (Equation 15). This approach does not require a complex two-state receptor model and could be used directly linked with existing BCPMs. While the novel approaches yield comparable results overall, the original model’s inability to incorporate pulsatile characteristics becomes evident in the case of glucocorticoid-induced osteoporosis, where the pulsatile pattern is characterized by increased relative amplitude without significant changes in maximum concentration (Figure 7). The original model, which neglects these pulsatile dynamics, fails to capture the catabolic cell responses and subsequent bone loss (Figures 9, 10). This addresses fundamental challenges in mathematical modeling of endocrine systems, where dynamic hormone secretion patterns are often oversimplified as constant values. The importance of capturing hormonal pulsatility is particularly evident in PTH signaling, where pulsatile *versus* continuous administration produces opposing effects on bone metabolism (Veldhuis, 2008). Our semi-coupled model incorporates both pulsatile characteristics and cellular desensitization through the two-state receptor model, addressing key research gaps identified in endocrine system modeling (Brucker-Davis et al., 2001; Zavala et al., 2019; Zavala, 2022).

The new model qualitatively predicts expected catabolic responses for the majority of PTH-driven bone diseases (hyperparathyroidism, hypocalcemia, glucocorticoid-induced osteoporosis), specifically, both the increased osteoclast activity and the resulting bone volume loss that characterize these conditions. It does not predict the bone loss encountered in osteoporosis and postmenopausal osteoporosis, where the model maintains homeostasis. The reason for this might be that these diseases are not exclusively linked to alterations in PTH release patterns, but also involve more significant pathophysiological changes such as estrogen depletion in PMO which directly regulates RANKL production by osteoblasts and osteocytes and/or TGF- β activity in old-age OP (Hawkins et al., 2021; Lu and Tian, 2023; Ruiz-Lozano et al., 2024). This is supported by the respective PTH characteristics (Table 1) that do not deviate significantly from the healthy pattern, confirming our model behaves as expected: predicting bone loss when PTH deviations are large enough compared to the healthy pattern while remaining stable when PTH patterns remain relatively normal despite the presence of other pathological mechanisms driving bone loss.

Validating model predictions against clinical data presents inherent challenges, as bone loss measurements typically compare disease states to controls rather than tracking progression from onset. For bone volume predictions (Figure 10), our model shows conservative estimates across conditions: For glucocorticoid-induced osteoporosis, we predict 0.7% trabecular bone loss in the first 60 days compared to clinical observations of approximately 5% loss (Boling, 2004). In primary hyperparathyroidism, our predicted loss of 0.8% after 60 days could reasonably accumulate to the observed 4%–5% difference between PHPT and control subjects (Christiansen et al., 1992), considering more rapid initial bone loss. The model’s highest catabolic response occurs in hypocalcemia, consistent with experimental data showing substantial bone loss (19% in rat models after 6 months (Jakubas-Przewłocka and Przewłocki, 2005), though our predicted magnitude is lower. These discrepancies likely reflect fundamental differences in disease simulation approaches (pulsatile human PTH patterns *versus* induced disease states), experimental duration, species transferability, and the instantaneous disease onset in our model *versus* progressive development in biological systems. While these comparisons suggest the need for parameter optimization based on expected bone loss, the heterogeneity and limited availability of consistent clinical data currently constrains such validation efforts, a challenge that has motivated various cross-methodological data-driven calibration approaches in bone modeling (Araujo et al., 2014; Baratchart et al., 2022).

Similar challenges apply to validating bone cell concentration predictions (Figures 8, 9), as osteoblasts and osteoclasts are rarely tracked over time in clinical settings. Comparison with murine studies helps establish reasonable numerical expectations despite inherent species differences. For hyperparathyroidism induced in mice, Siddiqui et al. (2017) demonstrated a 2.3-fold increase in osteoclast number per bone surface, while our model predictions range from 2.25 to 3.9-fold increases in OCa concentration across different approaches, showing good agreement with experimental data. This alignment is further supported by the 3.3-fold increase in TRAP5b–an enzyme produced by osteoclasts–observed in the same study. Our predicted decrease in bone volume fraction (0.2%–0.37%) is conservative compared to the experimental 9% reduction in the cortex, despite reasonable osteoclast predictions and only moderate underestimation of osteoblast activity (1.7–2.4-fold vs 3.67-fold experimental increase). Notably, no significant trabecular BV/TV loss was observed experimentally, which is consistent with our conservative bone volume estimates. For glucocorticoid-induced osteoporosis, Hofbauer et al. (2009) reported a 1.55-fold increase in osteoclast number per bone surface, closely matching our novel model formulations (1.62–1.67-fold increase of OCa-concentration), while the original approach showed insufficient response (1.06-fold increase). For osteoblast dynamics, our model simulates an initial decrease in active osteoblast concentration, which aligns qualitatively with the reduced serum osteocalcin levels reported for GIO-induced mice (Hofbauer et al., 2009). The initial decrease after disease onset is followed by a compensatory increase in response to elevated osteoclast activity. While this cellular response pattern is phenomenologically correct, we acknowledge that GIO involves complex mechanisms beyond PTH pulsatility alterations that our model does not capture, as our primary objective was demonstrating the importance of pulsatile hormone dynamics rather than comprehensive GIO modeling. Despite this cellular-level agreement, our model again predicted minimal bone volume changes over 80 days compared to substantial experimental bone area loss over 4 weeks (Hofbauer et al., 2009). Hofbauer et al. reported that trabecular BMD remained unchanged in GIO-induced mice, with most pronounced decreases in cortical and subcortical compartments. Likely due to limited sample sizes, BMD effects did not reach statistical significance. The agreement of our cellular predictions (osteoclasts and osteoblasts) with experimental data, coupled with underestimation of corresponding bone loss, suggests that recalibrating bone formation and resorption rate constants with appropriate human data could improve structural predictions. While these rates are currently assumed constant following established BCPM practice (Lemaire et al., 2004; Pivonka et al., 2008; Scheiner et al., 2013), experimental studies have demonstrated variability in individual osteoclast resorptive activity (Kanehisa and Heersche, 1988), and mathematical modeling of injury repair has provided *in vivo* evidence for time-variable cellular activity rates (Lo et al., 2021). Careful consideration is needed since our model tracks average cellular concentrations and thus inherently represents averaged resorption and formation rates over cell populations. Nevertheless, the order-of-magnitude agreement in cellular responses across both conditions provide confidence in the model’s mechanistic foundation.

We acknowledge several key limitations of our current approach. First, PTH levels are prescribed externally rather than evolving from endogenous physiological feedback mechanisms in both the original and our novel BCPM. PTH is either maintained at constant levels (original model) or follows prescribed pulsatile patterns (novel approach), without incorporating the calcium-PTH feedback loop that naturally regulates PTH secretion *in vivo* (Chiavistelli et al., 2015). This approach is appropriate for our primary objective of studying how specific hormone patterns affect bone cell dynamics and remodeling activity under controlled conditions. However, an autonomous model–incorporating calcium homeostasis and PTH regulation through calcium-sensing receptors–would facilitate analysis of the underlying causes of dysregulated hormone patterns themselves. An autonomous model would eliminate the current assumption of instantaneous disease state onset and allow gradual disease progression through feedback dysregulation.

Second, we model disease states as immediate transitions from a healthy PTH pattern (Figures 8, 9). This type of approach was original suggested by Lemaire et al. (2004) and subsequently improved by Pivonka et al. (2008) to account for temporal changing disease patterns. Our current approach could be extended towards gradual changing PTH patterns over given time intervals. Additionally, changes in bone volume fraction are constant after steady-states of cell concentration are reached. This implies constant bone loss independent of disease duration, which is not physiological. Mechanostat model incorporation could address this limitation (Scheiner et al., 2013; Martínez-Reina and Pivonka, 2019).

Third, our square-wave pulses for the PTH secretion pattern based on the original formulations (Li and Goldbeter, 1989; Martonová et al., 2023) represent an idealized version of hormone release patterns. A more physiologically realistic approach would model PTH degradation as an exponential decrease after the onset of each pulse, rather than an immediate switch to zero concentration, while maintaining total secreted PTH.

Finally, while we focused on PTH1R signaling in osteoblasts, recent studies have found that PTH1R is also expressed on osteocytes, where PTH directly induces upregulation of RANKL gene production. The resulting increased RANKL/OPG ratio leads to higher osteoclast recruitment and activation. This is consistent with the conservative bone loss predicted by the model. Additionally, PTH binding to PTH1R expressed on osteocytes downregulates sclerostin production, a formation inhibitor, which thus enhances bone formation (Ben-awadh et al., 2014; Marino and Bellido, 2024). These osteocyte-mediated effects represent additional pathways through which PTH influences bone remodeling beyond the osteoblast responses captured in our current model.

These limitations suggest several research directions. The framework could be extended to more sophisticated bone cell population models distinguishing between modeling and remodeling processes (Trichilo et al., 2019). The activity function α(t) could potentially distinguish between catabolic and anabolic pathways directly, making explicit, separate pathways unnecessary. Future developments could explore gradual concentration changes between healthy and disease states or directly use the time-dependent cellular activity function α(t) instead of constant PTH effect quantification.

Beyond PTH dynamics, our approach offers a template for other biological contexts where both temporal patterns and receptor adaptation require consideration. The methodology applies to mechanical loading patterns during habitual movement or exercise, where cells respond to pulsatile mechanical stimuli and adapt to sustained loads. This framework could also be adapted to other signaling pathways such as RANKL-RANK-OPG binding. This demonstrates the broader applicability in modeling various biological regulatory systems beyond simple Hill functions and constant stimuli. The interaction of bone remodeling, calcium homeostasis, and PTH secretion represents an interesting approach for future model development that could bridge the gap between prescribed hormone patterns and the physiological mechanisms that generate them.

## 5 Nomenclature

### 5.1 Resource identification initiative

All simulations were performed using Python Programming Language (RRID:SCR_008394).

## Data Availability

The datasets presented in this study can be found in online repositories (Modiz, 2025). The names of the repository/repositories and accession number(s) can be found below: https://github.com/cmodiz/Bone-Models.git.
