# Supplementary material for: Computational simulations of endocrine bone diseases related to pathological glandular PTH secretion using a multi-scale bone cell population model
Source: Front Bioeng Biotechnol. 2025 Oct 1;13:1619276. doi: 10.3389/fbioe.2025.1619276 (PMC12521151; doi:10.3389/fbioe.2025.1619276)
Supplement: Supplementary file 1 [file DataSheet1.pdf]

Supplementary Material

1 SUPPLEMENTARY DATA

To improve readability, the bone cell population model and the two-state receptor model are only described to a certain detail. The model definition and parameters are completed in the following.

The effect of RANKL-RANK binding, summarized in Eq. (S1), is given by the Hill-type function

$$\pi_{RANKL} = \frac{k_{RL}}{k_{-RL}} \left(\frac{K_L^{max} OB_a \pi_{PTH}}{1 + \frac{k_{RL} K_R}{k_{-RL}} + \frac{k_{OL}}{k_{-OL} k_O} \cdot \left(\frac{K_O^{min} OB_p}{\pi_{PTH}} \right)} \right). \tag{S1}$$

The corresponding parameters and description are given in Table S2 Lemaire et al. (2004).

The description of the two-state receptor model Martonová et al. (2023) does not include the definition of the basal activity α_0 and the integrated activity of a step increase. A more detailed description is given in the source publications Martonová et al. (2023); Li and Goldbeter (1989). The basal activity α_0 is defined as

$$\alpha_0 = \frac{1}{1 + \frac{k_{-1}}{k_1}} a_1 \frac{k_{-1}}{k_1} + a_4. \tag{S2}$$

The parameters for the two-state receptor model, can be found in Table S1. An exception is the activity constant a_2 given as

$$a_2 = \left(\frac{(a_1 \cdot K_1 + a_4) \cdot (K_2 + 1)}{(K_1 + 1)} - a_3 \right) \frac{1}{K_2}, \tag{S3}$$

with $K_1 = \frac{k_{-1}}{k_1}$ and $K_2 = \frac{k_{-2}}{k_2}$. The integrated activity of a step increase can be explicitly computed according to Li and Goldbeter (1989) as

$$\alpha_{T_{step}} = \tau_a \alpha_{M_{step}} \quad \text{with} \quad \tau_a = \frac{1}{u_1 + v_1} \quad \text{and} \quad \alpha_{M_{step}} = Q \cdot P. \tag{S4}$$

It holds for the first term that

$$u_1 = \frac{k_1 + k_2(\gamma_{on} + \gamma_{off})}{1 + \gamma_{on} + \gamma_{off}} \quad \text{and} \quad v_1 = \frac{k_{-1} + k_{-2}(\gamma_{on} + \gamma_{off})c}{1 + (\gamma_{on} + \gamma_{off})c}. \tag{S5}$$

For the second term

$$Q = D_{s_1} - D_{s_0} \quad \text{and} \quad P = A - B, \tag{S6}$$

with

$$u_0 = \frac{k_1 + k_2 \cdot \gamma_{off}}{1 + \gamma_{off}} \quad \text{and} \quad v_0 = \frac{k_{-1} + k_{-2} \cdot \gamma_{off} \cdot c}{1 + \gamma_{off} \cdot c}, \tag{S7}$$

$$D_{s_0} = u_0 \cdot \frac{1}{u_0 + v_0}, \quad \text{and} \quad D_{s_1} = u_1 \cdot \frac{1}{u_1 + v_1}, \tag{S8}$$

$$A = \frac{a_1 + a_2 \cdot (\gamma_{on} + \gamma_{off})}{1 + \gamma_{on} + \gamma_{off}}, \quad \text{and} \quad B = \frac{a_4 + a_3 \cdot (\gamma_{on} + \gamma_{off}) \cdot c}{1 + (\gamma_{on} + \gamma_{off}) \cdot c}, \tag{S9}$$

$$c = \frac{K_1}{K_2}. \tag{S10}$$

The kinetic parameters k_1, k_{-1}, k_2, k_{-2} are adopted from Martiel and Goldbeter for cAMP signaling Martiel and Goldbeter (1987), following the approach of Martonova et al. who justified this choice based on the similarity of GPCR two-state kinetics between systems and the technical challenges of directly measuring these parameters for PTH1R Martonová et al. (2023). The parameters $K_3 = k_{-3}/k_3$ are determined from the dissociation binding constant for PTH to PTH1R Yasuoka et al. (1996); Hoare and Usdin (1999), while $K_4 = k_{-4}/k_4$ is derived from the condition of detailed balance as prescribed in Li and Goldbeter Li and Goldbeter (1989). Despite the cross-system parameter adoption, our modeling framework remains robust to moderate parameter variations through calibration scaling during semi-coupling, ensuring that relative ratios between cellular responsiveness states are preserved across healthy and disease conditions.

Parameter	Value	Unit	Type
k_1	1.2e-2	1/min	Kinetic constant
k_{-1}	1.04e-1	1/min	Kinetic constant
k_2	2.22e-1	1/min	Kinetic constant
k_{-2}	5.5e-2	1/min	Kinetic constant
$K_3 = k_{-3}/k_3$	1	nM	Dissociation constant for active complex
$K_4 = k_{-4}/k_4$	10^3	nM	Dissociation constant for inactive complex
a_1	$s \cdot 20$	-	Activity constant
a_3	$s \cdot 10$	-	Activity constant
a_4	$s \cdot 1$	-	Activity constant
s	100	-	Scaling constant

Table S1. Kinetic and activity parameters for the two-state PTH receptor model, including rate constants $k_{\pm j}$ with $j \in \{1, 2, 3, 4\}$ and activity-related parameters from Martonová et al. (2023).

REFERENCES

Hoare, S. R. and Usdin, T. B. (1999). Quantitative cell membrane-based radioligand binding assays for parathyroid hormone receptors. *Journal of Pharmacological and Toxicological Methods* 41, 83–90. doi:https://doi.org/10.1016/S1056-8719(99)00024-6

Lemaire, V., Tobin, F. L., Greller, L. D., Cho, C. R., and Suva, L. J. (2004). Modeling the interactions between osteoblast and osteoclast activities in bone remodeling. *J. Theor. Biol.* 229, 293–309. doi:https://doi.org/10.1016/j.jtbi.2004.03.023

Li, Y. and Goldbeter, A. (1989). Frequency specificity in intercellular communication. influence of patterns of periodic signaling on target cell responsiveness. *Biophys. J.* 55, 125–145. doi:https://doi.org/10.1016/S0006-3495(89)82785-7

Parameter	Value	Unit	Description
D_{OB_u}	7.00e-4	pM/day	Differentiation rate of uncommitted osteoblasts
D_{OB_p}	$f_0 \cdot d_{OB_p}$	1/day	Differentiation rate of osteoblast precursors
d_{OB_p}	7.00e-1	1/day	Differentiation rate of osteoblast precursors without proportionality constant
f_0	5.00e-2	-	Fixed proportion for $\pi_{TGF-\beta}$
D_{OC_p}	2.10e-3	pM/day	Differentiation rate of osteoclast precursors
A_{OB_a}	1.89e-1	1/day	Apoptosis rate of active osteoblasts
A_{OC_a}	7.00e-1	1/day	Apoptosis rate of active osteoclasts
C_{PTH}^{basal}	250	pM/day	Rate of synthesis of systemic PTH
C_{PTH}^{inj}	0	pM/day	Externally injected PTH
k_1	2.00e-2	1/(pM day)	Rate of PTH-PTH1R binding
k_{-1}	3.00e+0	1/day	Rate of PTH-PTH1R unbinding
k_{RL}	5.80e-4	1/(pM day)	Rate of RANKL-RANK binding
k_{-RL}	1.70e-2	1/day	Rate of RANKL-RANK unbinding
k_{OL}	10e-2	1/(pM day)	Rate of RANKL-OPG binding
k_{-OL}	10e+0	1/day	Rate of RANKL-OPG unbinding
k_{PTH}	86	1/day	PTH elimination rate
k_O	3.5e-1	1/day	OPG elimination rate
$K_{TGF-\beta}$	5.00e-3	pM	Dissociation binding constant for $TGF-\beta$ to its receptor
K_R	10e+0	pM	Fixed concentration of RANK
K_L^{max}	3.00e+6	pM/pM cells	Maximum number of RANKL attached on each cell surface
K_O^{min}	2e+5	pM/pM cells	Minimal rate of production of OPG per cell
K_O^{min}	2e+5	pM/pM cells	Minimal rate of production of OPG per cell
t_{end}	140	days	End time for simulation
t_0^{LC}	20	days	Start time for disease state
t_{end}^{LC}	80	days	End time for disease state
k_{form}	0.01571	1/(pM day)	Relative rate of bone formation

Table S2. Table of parameters, values, units, and descriptions for the bone cell population model. The values are taken from the source publication Lemaire et al. (2004).

Martiel, J.-L. and Goldbeter, A. (1987). A model based on receptor desensitization for cyclic amp signaling in dictyostelium cells. *Biophysical Journal* 52, 807–828. doi:10.1016/S0006-3495(87)83275-7

Martonová, D., Lavaill, M., Forwood, M. R., Robling, A., Cooper, D. M. L., Leyendecker, S., et al. (2023). Effects of pth glandular and external dosing patterns on bone cell activity using a two-state receptor model—implications for bone disease progression and treatment. *PLoS One* 18, e0283544. doi:https://doi.org/10.1371/journal.pone.0283544

Yasuoka, T., Kawashima, M., Takahashi, T., Iwata, A., Oka, N., and Tanaka, K. (1996). Changes in parathyroid hormone receptor binding affinity during egg laying: Implications for calcium homeostasis in chicken. *Journal of Bone and Mineral Research* 11, 1913–1920. doi:https://doi.org/10.1002/jbmr.5650111212
